# Appearance of New Cutaneous Superficial Basal Cell Carcinomas during Successful Nivolumab Treatment of Refractory Metastatic Disease: Implications for Immunotherapy in Early Versus Late Disease

**DOI:** 10.3390/ijms18081663

**Published:** 2017-07-31

**Authors:** Philip R. Cohen, Shumei Kato, Aaron M. Goodman, Sadakatsu Ikeda, Razelle Kurzrock

**Affiliations:** 1 Department of Dermatology, University of California San Diego, San Diego, CA 92122, USA; 2 Department of Medicine, Division of Hematology/Oncology, University of California San Diego, La Jolla, CA 92093, USA; smkato@ucsd.edu (S.K.); a1goodman@ucsd.edu (A.M.G.); saikeda@ucsd.edu (S.I.); rkurzrock@ucsd.edu (R.K.); 3 Center of Personalized Cancer Therapy, University of California San Diego, La Jolla, CA 92093, USA; 4 Department of Medicine, Division of Blood and Marrow Transplantation, University of California San Diego, La Jolla, CA 92093, USA; 5 Medical Hospital, Central Clinical Facilities, Cancer Center, Tokyo Medical and Dental University, Tokyo 113-8510, Japan

**Keywords:** basal, carcinoma, cell, cutaneous, immunotherapy, metastatic, nivolumab

## Abstract

Metastatic basal cell carcinoma may be treated with hedgehog pathway inhibitors, including vismodegib and sonidegib. However, patients can demonstrate resistance to these agents. We describe a man with metastatic basal cell carcinoma who did not respond well to vismodegib and sonidegib. Next generation sequencing of his metastatic liver tumor demonstrated a high tumor mutational burden (103 mutations per megabase) and the genomic amplification of *PD-L1*, both of which are features that predict response to anti-PD1/PD-L1 immunotherapy. Treatment with nivolumab, an anti-PD1 checkpoint inhibitor, resulted in near complete remission. Yet, he developed new primary cutaneous basal cell carcinomas while receiving immunotherapy and while his metastatic disease showed an ongoing response. His new superficial skin cancer had a lower tumor mutational burden (45 mutations per megabase) than the metastatic disease. Since immunotherapy response rates are higher in patients with more genomically complex tumors, our observations suggest that, in contrast with the premise of earlier treatment is better, which holds true for targeted and cytotoxic therapies, immunotherapy may be better suited to more advanced disease.

## 1. Introduction

Metastatic basal cell carcinoma is uncommon [1]. Vismodegib and sonidegib are targeted therapies that act on the Hedgehog pathway. These agents can be effective in treating patients with locally advanced or metastatic basal cell carcinoma [2,3]. Individuals with metastatic basal cell carcinoma who develop resistance to these compounds may respond to other therapies that are targeted to specific genome aberrations of their tumors [4].

We describe a unique patient with metastatic basal cell carcinoma resistant to vismodegib and sonidegib who had an amplification of programmed death-ligand 1 (*PD-L1*, also called *CD274*), programmed death-ligand 2 (*PD-L2*, also called *PDCD1LG2*), and Janus kinase 2 (*JAK2*); he exhibited an exceptional response to anti-programmed death 1 (PD1) therapy [5]. Yet, subsequent to his ongoing near complete remission of his metastatic disease, he has developed new cutaneous basal cell carcinomas. A genomic evaluation of the tumors was performed and yields new insights into the effects of immunotherapy on early versus late disease.

## 2. Case Presentation

A 58-year-old man (fair skinned with a history of sun exposure) presented in 2016 for evaluation of newly appearing scaly red plaques on his left shoulder and left clavicle. His past medical history was significant for not only multiple primary cutaneous basal cell carcinomas, but also metastatic basal cell carcinoma with involvement of the brain, bone, liver, and lungs. The new skin lesions had appeared in the setting of near complete remission of his metastatic disease on continued nivolumab treatment; the successful management of his metastatic disease—prior to the onset of his recently developed cutaneous tumors—has previously been described [5]. This study was performed and consents obtained in accordance with UCSD IRB guidelines (NCT02478931).

The history of the present illness reveals that, in 2012, he developed a basal cell carcinoma on his left posterior shoulder. The tumor was excised and recurred. The subsequent postoperative wound margins were positive for carcinoma, and the site was treated with radiotherapy. Two years later, in 2014, an evaluation of back pain revealed metastatic basal cell carcinoma not only to his axial skeleton, but also to his liver and lungs. The diagnosis was confirmed with biopsies from the bone and liver. A soft tissue specimen of metastatic tumor also demonstrated basal cell carcinoma.

In August 2014, he started vismodegib. The treatment was discontinued after three months when he developed brain metastases. Following successful stereotactic radiosurgery, he began cisplatin and paclitaxel in November 2014. In March 2015, after four cycles of chemotherapy, the drugs were discontinued secondary to progressive bone and liver metastatic disease. Sonidegib combined with buparlisib (a pan-class I PIK3 inhibitor) was started in May 2015, and was discontinued six weeks later after disease progression in the liver. He received vismodegib and paclitaxel from July 2015 to September 2015; they were discontinued secondary to a lack of response.

Hybrid capture-based next generation sequencing (236 genes) was performed by Foundation Medicine on a liver tumor biopsy specimen from July 2015 (Table 1; Available online: http://www.foundationone.com/) [6]. The sequencing demonstrated a tumor mutational burden of 103 mutations per megabase (reference: >19 mutation per megabase = high tumor mutation burden) and multiple genomic alterations, including an amplification of *PD-L1*, *PD-L2*, and *JAK2*. In October 2015, based on the high tumor mutational burden and *PD-L1* amplification, both of which are associated with a response to checkpoint inhibitors, he was started on the checkpoint inhibitor nivolumab at 240 mg intravenously every 2 weeks, with a remarkable and rapid improvement of performance status, and tumor shrinkage to near complete remission.

**Figure 1:**
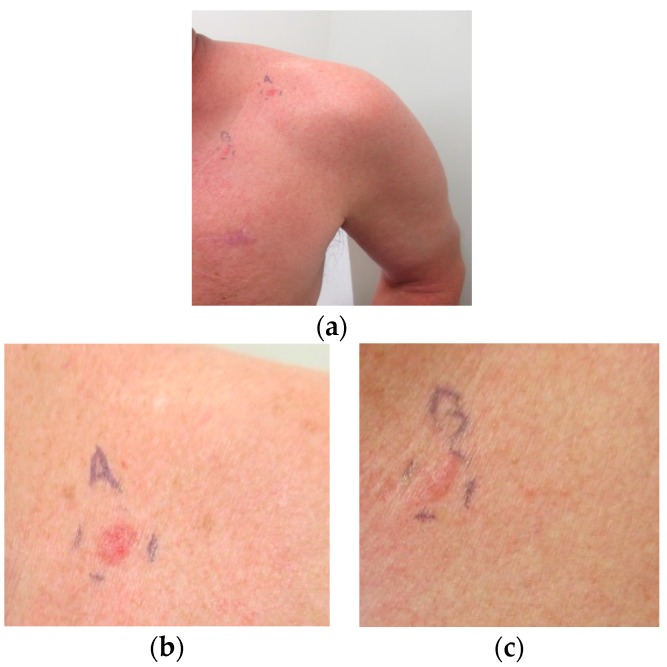
Superficial basal cell carcinomas presenting as erythematous plaques on the left anterior shoulder (labeled A) and the left chest (labeled B) in a man whose metastatic basal cell carcinoma is being treated with a checkpoint inhibitor and is in near complete remission; (**a**) closer views of the new primary skin cancers on the left anterior shoulder (**b**) and left chest (**c**) that developed while the patient’s metastatic basal cell carcinoma was responding to nivolumab. The patient gave signed informed consent for data analysis and the publication of the images.

**Figure 2:**
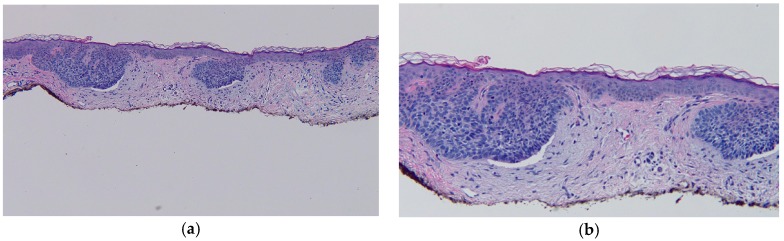
Distant (**a**) and closer (**b**) views demonstrate the microscopic features of the left anterior shoulder superficial basal cell carcinoma. There is orthokeratosis overlying an atrophic epidermis with flattening of the rete ridges. Superficial buds of basaloid tumor cells extend from the epidermis into the papillary dermis. There is palisading of the tumor keratinocytes at the periphery of the aggregates of carcinoma and retraction of the surrounding dermal stroma resulting in cleft formation. There is solar elastosis, small telangiectasias, and a sparse lymphocytic inflammatory infiltrate (hematoxylin and eosin: **a**, 4×; **b**, 10×).

He presented with new erythematous plaques on his left anterior shoulder (6 mm × 6 mm) and left chest (8 mm × 6 mm) in June 2016 (Figure 1). He was still receiving nivolumab, and the near complete remission of his metastatic basal cell carcinoma was ongoing. Biopsy specimens from both skin lesions showed similar pathologic changes, confirming the diagnosis of superficial basal cell carcinoma: superficial buds of basaloid tumor cells extending from the overlying epidermis into the dermis (Figure 2).

Next generation sequencing of the specimen from his left anterior shoulder primary cutaneous basal cell carcinoma was performed (Table 1). The sequencing demonstrated a tumor mutational burden of 45 mutations per megabase and eight characterized genomic alterations. In contrast to the metastatic basal cell carcinoma in his liver, the primary skin cancer did not demonstrate amplification of *PD-L1*, *PD-L2* or *JAK2*.

The superficial basal cell carcinomas on his left anterior shoulder and left chest were each treated with electrodessication and curettage. A follow up examination in February 2017 showed complete healing of the treated skin cancer sites without tumor recurrence. He was still receiving nivolumab every other week, and his metastatic basal cell carcinoma continued to demonstrate over 95% regression on imaging.

## 3. Discussion

Basal cell carcinoma is the most common skin cancer [7]. It is frequently associated with *PTCH1* gene aberrations [8]. Metastatic basal cell carcinoma may be responsive to agents such as vismodegib and sonidegib directed toward the Hedgehog pathway. There are anecdotal reports of success with other therapies targeted to tumor-specific genomic aberrations [2,3,4].

Checkpoint inhibitors, such as nivolumab, may be effective immunotherapy agents for patients with increased PD-L1 expression [9,10,11]. *PD-L1* amplification (as seen in this patient’s metastatic liver tumors) may be an especially strong predictor of response to anti-PD1/PD-L1 drugs [9,10,11,12,13]. This association has been demonstrated in patients with heavily pretreated Hodgkin lymphoma, a disease whose hallmark is *PD-L1* amplification; response rates to anti-PD1 agents are in the range of 65 to 85% [11,12,13]. Tumors with multiple genomic aberrations (increased tumor mutation burden) also have a higher chance of producing immunogenic neo-antigens; therefore, they too may be highly responsive to anti-PD1 therapy [9,10].

The reported patient with metastatic basal cell carcinoma was resistant to the genomically targeted therapies vismodegib and sonidegib. The lack of response to therapy targeting a single genomic aberration is not surprising in light of the fact that the patient’s metastatic tumor had numerous alterations (total = 19 characterized alterations in genes known be important in cancer) (Table 1). Indeed, previous data have suggested that response to genomically targeted therapy is inversely proportional to the number of alterations in the tumor [14]. As a corollary, early disease may be much more responsive to genomically targeted therapy than late disease. Chronic myelogenous leukemia (CML), whose hallmark is the aberrant *BCR-ABL* fusion, exemplifies this phenomenon. With the Bcr-Abl targeted drug imatinib, long-lasting responses are achieved in most newly diagnosed patients, but the durable response rate is negligible in late-stage disease [15]. This dichotomy is presumably due to clonal molecular evolution and an increasing mutational burden with disease progression.

As a general principle, tumors evolve with time and become genomically more complex. However, the question then arises as to whether or not the paradigm of “earlier treatment is better” applies to immunotherapy, since immunotherapy is more effective in more genomically complex disease with a higher mutational burden [9]. Therefore, since later disease is more genomically complex, it is conceivable that more advanced disease will be more responsive to immunotherapy. Indeed, it is intriguing that Richter’s transformation of chronic lymphocytic leukemia (CLL) appears to be significantly more responsive to an immune checkpoint blockade than earlier-stage CLL [16]. Theoretically, the better responses in the more advanced Richter’s transformation could be due to greater genomic complexity. Even so, other factors (a viral underpinning for Richter’s (since some virally associated tumors respond better to checkpoint inhibitors) or greater use of the immunosuppressive agent fludarabine in early CLL) cannot be ruled out as a cause for the discordant responsiveness. Our current observations, however, also support the idea that patients with later disease do better with immunotherapy, as our patient’s metastatic disease showed an ongoing near-complete response, even as new primary basal cell carcinomas that were significantly less genomically complex (and with low tumor mutational burden) than the metastatic disease appeared (Table 1). Of further interest in this regard, it has been demonstrated that, in basal cell carcinomas, the expression of PD-L1 increases with the number of prior treatment modalities [17]. The inactivation of the immune system via high PD-L1 expression may be necessary for a tumor with a high mutational burden to evade eradication by the immune system, and tumors with high PD-L1 are also more likely to respond to anti-PD1/PD-L1 agents.

The metastatic liver and subsequent primary skin basal cell carcinoma in our patient had a common genomic alteration of *CTNNA1* R383H. The same molecular aberration was also observed on a specimen of basal cell carcinoma obtained from soft tissue in 2014 prior to his receiving systemic chemotherapy [5]. A coincidental mutation in the same locus of the same gene in all three tumors seems unlikely. Therefore, the unifying molecular aberration raises the possibility of a germline mutation in the *CTNNA1* gene. To date, germline mutations in this gene have been reported anecdotally in hereditary gastric cancer [18].

## 4. Conclusions

Targeted therapies, such as vismodegib and sonidegib aimed at the Hedgehog pathway, are effective agents for treating patients with metastatic basal cell carcinoma. However, patients whose tumors develop resistance to these drugs have few options. There are now reports that they may be successfully treated with checkpoint inhibitors such as nivolumab [5]. Our patient’s metastatic tumor developed additional characterized genomic aberrations and a higher tumor mutational burden as his disease progressed. Of special interest was the observation that, in the setting of “late” metastatic disease, the tumor was highly susceptible to anti-PD1 immunotherapy. However, he continued to develop new primary cutaneous basal cell carcinomas even though he was receiving nivolumab and his metastatic disease remained in near complete remission. The checkpoint inhibitor did not prevent his new localized superficial basal cell carcinomas in the skin, perhaps since the “early” primary disease had a lower tumor mutational burden. In summary, immunotherapy may be best suited for “late” disease, in which the metastatic tumor has many genomic aberrations and an increased tumor mutational burden, in contrast to “early” disease, in which the primary cancer has fewer molecular alterations and a lower tumor mutational burden. There is a need for prospective studies to investigate the general role of immunotherapy in early versus late disease.

## Figures and Tables

**Table 1:** Genomic aberrations of patient’s basal cell carcinomas.

Genomic Alteration	Soft Tissue Tumor (2014)	Liver Metastasis (2015)	New Primary Cutaneous Tumor that Appeared while His Metastatic Disease was Responding to Nivolumab (2016)
*AXIN1* T601		No	Yes
*BAP1* K368 *		No	Yes
*CARD11* E756K		No	Yes
*CDKN1A* R140Q	Yes	Yes	No
*CDKN2A p16INK4a* P81L	Yes	Yes	No
***CTNNA1* R383H**	**Yes**	**Yes**	**Yes**
*FLT1* E487K		Yes	No
*JAK2* amplification		Yes	No
*KDR* R1032Q		No	Yes
*LRP1B* splice site 9121-1G > A	Yes	Yes	No
*LRP1B* W2334 *		Yes	No
*MLL2* splice site 4132-1G > A		Yes	No
*NOTCH1* W287 *	Yes	Yes	No
*PDGFRA* E459K		Yes	No
*PD-L1 (CD274)* amplification		Yes	No
*PD-L2 (PDCD1LG2)* amplification		Yes	No
*PIK3R2* Q412 *		Yes	No
*PTCH1* Q1366 *	Yes	Yes	No
*PTCH1* S181 *, splice site 584+1G > A		No	Yes
*PTCH1* W197 *	Yes	Yes	No
*SLIT2* K325 *	Yes	Yes	No
*SMARCA4* Q1166 *	Yes	Yes	No
*SPEN* R1854Q		No	Yes
*TERT* promoter-139-138CC > TT		Yes	No
*TP53* E285K		No	Yes
*TP53* P278S	Yes	Yes	No
Total Characterized alterations	10	19	8
Tumor mutation burden (mutations/megabase)	79	103	45

Abbreviations: **bold**, possible germ cell mutation.
